# Patterns of multiple brain network activation in dot perspective task

**DOI:** 10.1038/s41598-023-33427-1

**Published:** 2023-04-26

**Authors:** Marie-Louise Montandon, Cristelle Rodriguez, François R. Herrmann, Ariel Eytan, Alan J. Pegna, Sven Haller, Panteleimon Giannakopoulos

**Affiliations:** 1 Department of Psychiatry, Faculty of Medicine, University of Geneva, Geneva, Switzerland; 2 Department of Rehabilitation and Geriatrics, Geneva University Hospitals and University of Geneva, Geneva, Switzerland; 3 Division of Institutional Measures, Medical Direction, Geneva University Hospitals, Geneva, Switzerland; 4 School of Psychology, University of Queensland, Brisbane, Australia; 5 CIMC - Centre d’Imagerie Médicale de Cornavin, Geneva, Switzerland; 6 Department of Surgical Sciences, Radiology, Uppsala University, Uppsala, Sweden; 7 Faculty of Medicine of the University of Geneva, Geneva, Switzerland; 8 Department of Radiology, Beijing Tiantan Hospital, Capital Medical University, Beijing, China

**Keywords:** Empathy, Attention, Decision, Perception

## Abstract

In this functional MRI (fMRI) study on 82 healthy adults using the dot perspective task, inconsistency of perspectives was associated with a significant increase of the mean reaction time and number of errors both in Self and Other conditions. Unlike the Arrow (non-mentalizing), the Avatar (mentalizing) paradigm was characterized by the recruitment of parts of the mentalizing and salience networks. These data provide experimental evidence supporting the fMRI distinction between mentalizing and non-mentalizing stimuli. A widespread activation of classical theory of mind (ToM) areas but also of salience network and decision making areas was observed in the Other compared to Self-conditions. Compared to Self-Consistent, Self-Inconsistent trials were related to increased activation in the lateral occipital cortex, right supramarginal and angular gyrus as well as inferior, superior and middle frontal gyri. Compared to the Other-Consistent, Other-Inconsistent trials yielded strong activation in the lateral occipital cortex, precuneus and superior parietal lobule, middle and superior precentral gyri and left frontal pole. These findings reveal that altercentric interference relies on areas involved in self-other distinction, self-updating and central executive functions. In contrast, egocentric interference needs the activation of the mirror neuron system and deductive reasoning, much less related to pure ToM abilities.

## Introduction

Recent insights proposed that in neurotypical individuals there are two theory of mind (ToM) systems: one controlled that acts when we deliberately consider other’s thoughts and emotions and one implicit that involves the spontaneous analysis of their viewpoints even when such analysis is irrelevant for task processing^1–4^. Implicit ToM is thought to be developed early during development and remain stable over lifespan^3^. Although there is a wide agreement that adult humans are able to engage in unconscious analyses of others’ mental states^5^, several studies have led to conflicting data regarding the reliability of this concept using non-verbal measures such as violation of expectation paradigms, interactive and anticipatory looking tasks (for review see^6^). Spontaneous perspective taking is a facet of implicit ToM^7–9^. This human ability may be of key importance for the mostly unconscious ascription of mental states needed for social interactions. In a recent study, Drayton and colleagues demonstrated that psychopaths display significant deficits in their ability to take into account the other’s perspective that were associated with the number of their criminal convictions^10^. The dot perspective task (dPT) developed by Samson et al.^8^ has been used to examine the interference between self and an avatar perspective. Using this paradigm, prolonged reaction times were reported in case of divergent viewpoint both when focusing to our own perspective (altercentric interference) but also avatar’s perspective (egocentric interference)^8, 10^. Importantly, the contamination by the other’s perspective would take place spontaneously and occurs even when it is not pertinent for, and even a hindrance to, the achievement of our own goals. To date, there is still an ongoing theoretical debate of whether these interference effects in the dot-perspective task represent implicit mentalizing that would depend on the human nature of the Avatar or domain-general attention-orienting processes that would occur even when an arrow replaces the avatar form (for review see^11–14^).

Functional MRI (fMRI) studies attempted to explore the patterns of brain activation during the successful performance of Samson’s dPT with contradictory outcomes. Early observations showed an activation of the dorsolateral prefrontal and parietal cortices when selecting both one’s own over another’s visual perspective, and vice versa. Importantly, this activation decreased in the absence of conflict between the two viewpoints (consistent trials) when judging one’s own perspective, supporting the idea of a spontaneous mentalizing process^15^. In the same line, Schurz and collaborators^16^ reported a domain-specific activation in right temporo-parietal junction (TPJ), ventral medial prefrontal cortex (PFC) and ventral precuneus that occurs spontaneously in response to other’s perspective during the self-perspective judgements, although this position has been later challenged by a transcranial magnetic stimulation study that reported the stimulation of the right TPJ impaired performances even in control conditions which employed non mentalistic stimuli (arrows)^13^. In a meta-analysis of fMRI findings, Arora et al.^17^ reported activation overlap of visual perspective tasks with various ToM paradigms (false beliefs, trait judgment, rational action, social animation), but no consistent overlap with the ToM core regions such as the medial prefrontal cortex and bilateral posterior TPJ. Left TPJ/inferior parietal cortex as well as bilateral inferior frontal gyrus (for inhibiting one’s own perspective) were the only areas to be consistently activated in visual perspective taking paradigms. Referring to a large variety of neuroscientific methods (EEG, fMRI, near-infrared spectroscopy, transcranial direct current stimulation and transcranial magnetic stimulation), the review of Bukowski reported a regular involvement of frontal lobe areas (dorsolateral PFC, posterior middle and inferior frontal gyrus), dorsal precuneus and TPJ, as well as inferior parietal sulcus, inferior posterior temporal cortex and superior cerebellum^18^.

Despite the efforts to define the neural bases of visual perspective taking, methodological shortcomings should be considered. First, most fMRI studies were based on small samples and did not explore systematically the distinct role of parameters such as presence or absence of mentalizing processes (Avatar versus Arrow), self- versus other contrast, and consistency of perspectives. Only rare fMRI studies considered the effect of consistency in brain activation patterns and investigated the neural substrates of egocentric and altercentric interference in non-clinical populations^15, 16, 19^. In order to address these issues, we provide here a fMRI study in 82 healthy adults using Samson et al.’s experimental design^8^, while including Avatars and Arrows and analyzing activation patterns for self and other perspective taking conditions in both consistent and inconsistent trials.

## Results

### Behavioral data

Mean reaction times and the occurrence of errors for Arrow and Avatar, Self and Other and Consistent and Inconsistent conditions are summarized in Table 1.

**Table 1:** Mean reaction times (RT) presented as mean ± standard deviation (SD) (N = 1308 observations by cell) and number of errors (N = 1304 observations by cell).

	Inconsistent			Consistent			Total		
	Arrow	Avatar	Total	Arrow	Avatar	Total	Arrow	Avatar	Total
Other									
Mean RT	849.3	803.7	826.5	749.7	714.2	732	799.5	758.9	779.2
SD RT	377.8	333.1	356.8	295.5	266.9	282.1	342.8	305.1	325.1
Number of errors	35	35	70	14	13	27	49	48	97
Self									
Mean RT	810.9	808.7	809.8	758.1	742.7	750.4	784.5	775.7	780.1
SD RT	355.4	339.3	347.4	300.3	300.8	300.6	330	322.3	326.1
Number of errors	48	42	90	15	17	32	63	59	122
Total									
Mean RT	830.1	806.2	818.1	753.9	728.5	741.2	792	767.3	779.7
SD RT	367.2	336.2	352.2	297.9	284.6	291.6	336.5	313.9	325.6
Number of errors	83	77	160	29	30	59	112	107	219

N = 82 participants.

In a mixed model taking into account the three experimental conditions, there was a main effect of Avatar and Consistent conditions for mean reaction times whereas only Consistent condition was associated with the occurrence of errors. The only two significant interaction terms concerned Consistent # Self and Self # Avatar for mean reaction times (Table 2).

**Table 2:** Results of multiple mixed regression models with and without interaction terms (#): linear for mean reaction times (N = 10,464 observations) and logistic for errors (N = 10,432 observations).

	Reaction time			Errors		
	Coeff adjusted	95% CI	p-value	OR adjusted	95% CI	p-value
Model without interaction						
Consistent	− 76.96	[− 86.67, − 67.25]	< 0.001	0.35	[0.26, 0.47]	< 0.001
Self	0.89	[− 8.82, 10.60]	0.857	1.28	[0.97, 1.69]	0.080
Avatar	− 24.69	[− 34.40, − 14.98]	< 0.001	0.95	[0.72, 1.25]	0.726
Model with interactions						
Consistent	− 99.59	[− 118.99, − 80.20]	< 0.001	0.38	[0.20, 0.72]	0.003
Self	− 38.37	[− 57.77, − 18.98]	< 0.001	1.41	[0.90, 2.23]	0.137
Avatar	− 45.63	[− 65.03, − 26.24]	< 0.001	1.00	[0.61, 1.63]	1.000
Consistent # self	46.79	[19.36, 74.22]	0.001	0.76	[0.32, 1.81]	0.537
Consistent # avatar	10.15	[− 17.28, 37.57]	0.468	0.93	[0.37, 2.30]	0.869
Self # avatar	43.40	[15.97, 70.83]	0.002	0.86	[0.45, 1.66]	0.659
Consistent # self # avatar	− 23.33	[− 62.12, 15.46]	0.239	1.42	[0.42, 4.88]	0.574

N = 82 participants.

The mean reaction time for Arrow was significantly higher than that for Avatar. These values were similar for Self and Other conditions. However, there was a marked increase in mean reaction times in Self Inconsistent compared to Self Consistent and in Other Inconsistent compared to Other Consistent conditions. The occurrence of errors was similar in Arrow and Avatar, Self and Other conditions. As for mean reaction times, inconsistency was associated with a significant increase of the occurrence of errors both in Self and Other conditions (Table 3). These data confirm the presence of both egocentric and altercentric interference in the present sample as indicated previously ^8, 10^. Of importance, the effect of inconsistency was found both for the Avatar and Arrow conditions.

**Table 3:** Results of simple mixed regression models focusing on differences between the main conditions: linear for mean reaction times and logistic for errors.

	Reaction time			Errors		
	Coeff	95% CI	p	OR	95% CI	p
Arrow vs avatar	− 24.69	[− 34.51, − 14.87]	< 0.001	0.95	[0.73,1.25]	0.728
Self vs other	0.89	[− 8.94,10.72]	0.859	1.28	[0.97,1.68]	0.082
Consistent self vs inconsistent self (arrow + avatar)	− 59.39	[− 72.87, − 45.92]	< 0.001	0.33	[0.21,0.49]	< 0.001
Consistent self vs inconsistent self (arrow)	− 52.80	[− 72.03, − 33.58]	< 0.001	0.29	[0.16,0.53]	< 0.001
Consistent self vs inconsistent self (avatar)	− 65.99	[− 84.90, − 47.07]	< 0.001	0.36	[0.20,0.66]	0.001
Consistent other vs inconsistent other (arrow + avatar)	− 94.52	[− 108.39, − 80.65]	< 0.001	0.37	[0.24,0.58]	< 0.001
Consistent other vs inconsistent other (arrow)	− 99.59	[− 120.49, − 78.70]	< 0.001	0.39	[0.21,0.73]	0.003
Consistent other vs inconsistent other (avatar)	− 89.45	[− 107.53, − 71.37]	< 0.001	0.34	[0.18,0.66]	0.001

N = 82 participants.

### GLM analyses of task-related activation

**Figure 1:**
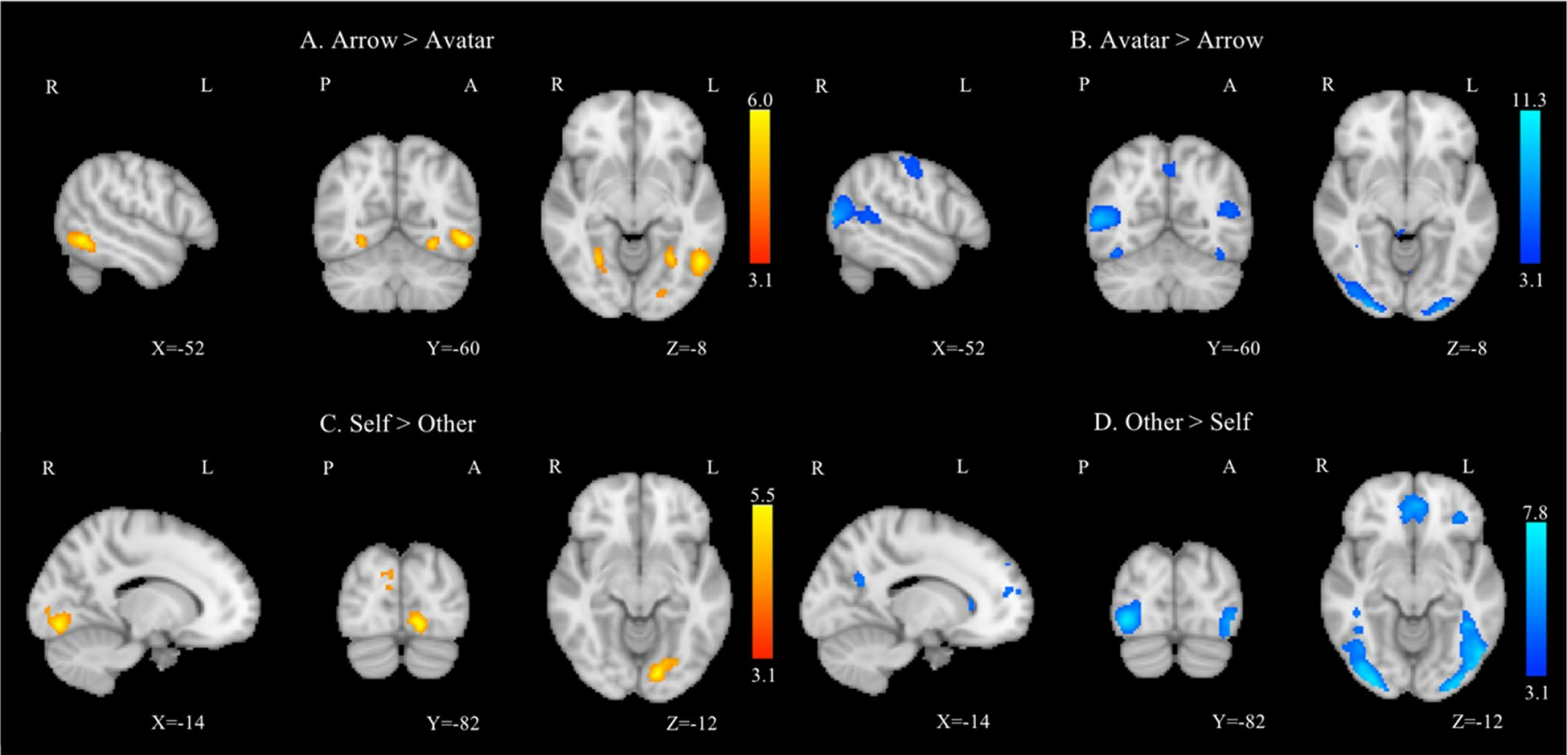
Group average task-related GLM analysis for the contrast Arrow > Avatar (**A**) and for the inverse comparison Avatar > Arrow (**B**), and for the contrasts Self > Other (**C**) and Other > Self (**D**). Right hemisphere on the left side, all clusters were thresholded by Z > 3.1 and a corrected cluster significance threshold of p = 0.05.

The activation clusters for each contrast with MNI coordinates of the cluster centers are provided as supplementary material (Tables 1–20). In the task-related GLM analysis, the comparison “Arrow versus Avatar” revealed increased activation in the bilateral lateral occipital and temporal occipital fusiform cortices. The inverse comparison yielded higher activation in the bilateral occipital pole and the left lateral occipital cortex, the bilateral precuneus cortex, the right angular and supramarginal gyri, the bilateral frontal pole, the anterior and posterior cingulate cortices (Fig. 1A,B). When considering these contrasts only for the Self Inconsistent condition, we observed an increased BOLD activation for Arrows confined to occipital areas. The “Avatar versus Arrow” contrasts led to additional activation in precuneus as well as superior and inferior frontal gyrus (See Supplementary Materials; Tables 18 and 19).

The comparison “Self versus Other” revealed strong BOLD activation in the left lingual and supramarginal gyri. Additional activations were present in the occipital cortex, notably the primary visual area, related to the visual stimulus presentation. The inverse comparison generated higher activation in the right lateral occipital and precuneus cortices, the left fusiform cortex, the left medial and orbitofrontal cortex, as well as the posterior cingulate gyrus (Fig. 1C,D).

**Figure 2:**
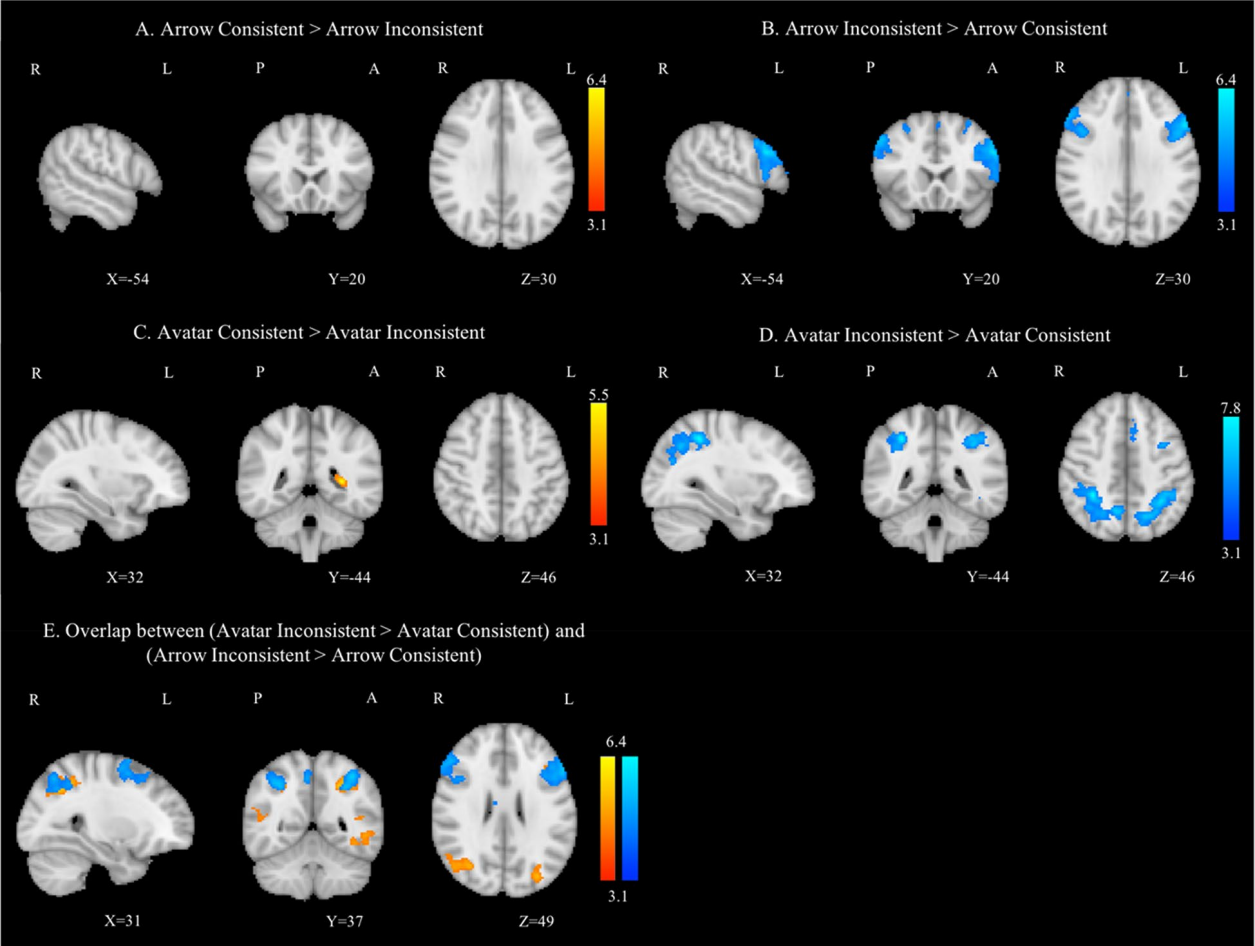
Group average task-related GLM analysis for the contrast Arrow Consistent > Arrow Inconsistent (**A**) and for the inverse comparison Arrow Inconsistent > Arrow Consistent (**B**), for the contrasts Avatar Consistent > Avatar Inconsistent (**C**) and Avatar Inconsistent > Avatar Consistent (**D**), and for the overlap between the contrasts Avatar Inconsistent > Avatar Consistent (in red) and Arrow Inconsistent > Arrow Consistent (in blue) (**E**). Right hemisphere on the left side, all clusters were thresholded by Z > 3.1 and a corrected cluster significance threshold of p = 0.05.

The contrast "Arrow Consistent versus Arrow Inconsistent" showed no activation cluster. The inverse contrast revealed an increased activation in the bilateral lateral occipital cortex, superior parietal lobule, middle and left superior frontal gyrus, and frontal pole (Fig. 2A,B). The average activation of the contrast "Avatar Consistent versus Avatar Inconsistent" revealed higher activation in the left lateral ventricle and cerebral white matter. The inverse contrast was more pronounced in the bilateral superior parietal lobule, the left middle and superior frontal gyri, and the left middle temporal gyrus (Fig. 2C–E). The Avatar Inconsistent – Avatar Consistent > Arrow Inconsistent – Arrow Consistent led to no cluster activation. The inverse contrast revealed higher activation only in lateral ventricles and caudate nuclei (Supplementary Materials; Table 20).

**Figure 3:**
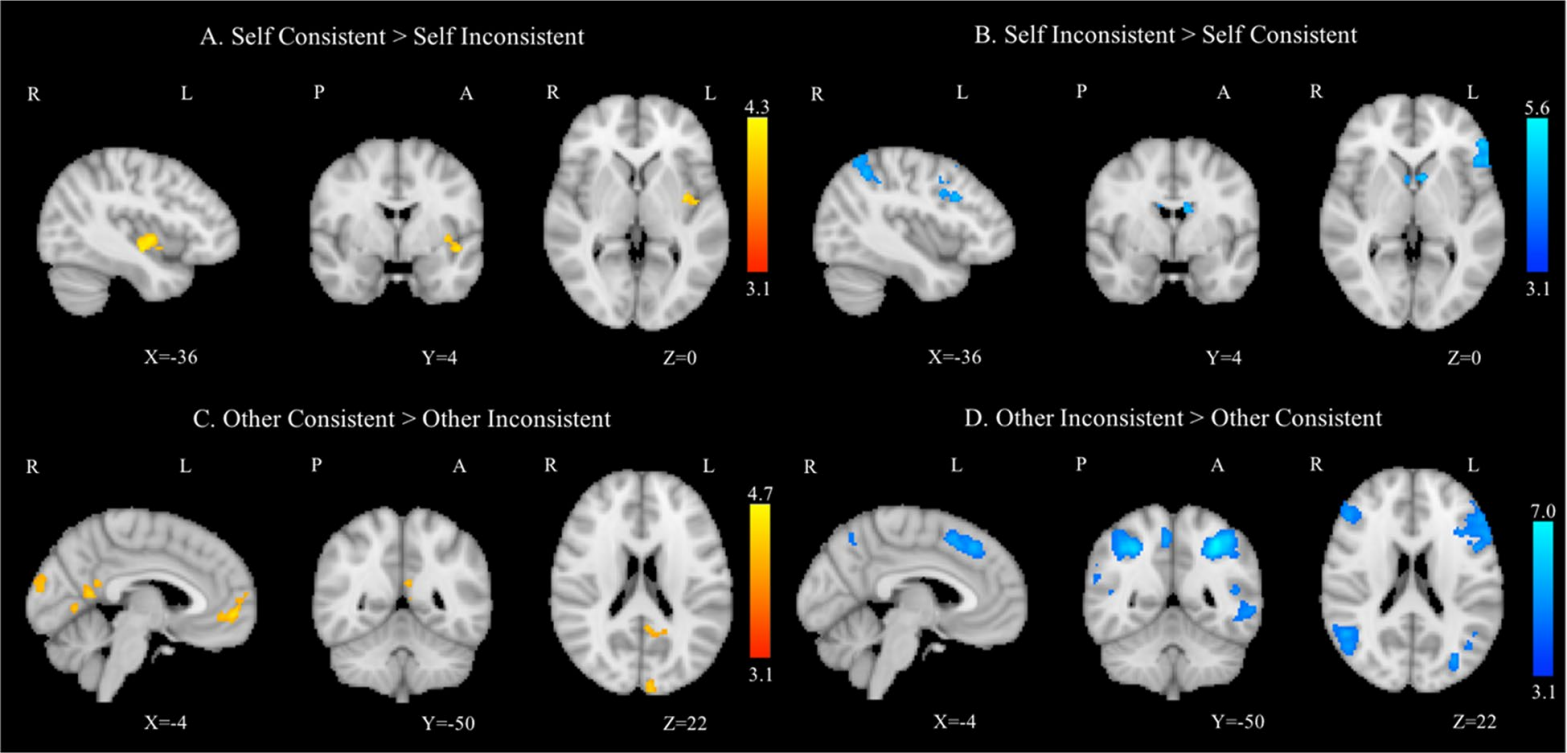
Group average task-related GLM analysis for the contrast Self Consistent > Self Inconsistent (**A**) and for the inverse comparison Self Inconsistent > Self Consistent (**B**), and for the contrasts Other Consistent > Other Inconsistent (**C**) and Other Inconsistent > Other Consistent (**D**). Right hemisphere on the left and side, all clusters were thresholded by Z > 3.1 and a corrected cluster significance threshold of p = 0.05.

The contrast “Self Consistent versus Self Inconsistent” showed and activation of the left insular cortex. The inverse contrast was more pronounced in the lateral occipital cortex bilaterally, the right supramarginal gyrus and the bilateral angular gyrus, and the bilateral inferior, superior and middle frontal gyri (Fig. 3A,B). Interestingly, when considering only the Avatars, this latter contrast led to increased BOLD activation in superior frontal and orbitofrontal cortex, inferior and middle temporal gyrus, supramarginal gyrus, superior parietal lobule and cingulate gyrus (see Supplementary Materials; Table 16). The average activation of the contrast “Other Consistent versus Other Inconsistent” yielded strong activation in the left occipital pole, precuneus cortex and lingual gyri, and in the right frontal medial cortex and anterior cingulate gyrus. The inverse contrast generated significantly increased BOLD activation in the bilateral lateral occipital cortex, precuneus cortex and superior parietal lobule, and in the middle, superior, precentral gyri and left frontal pole (Fig. 3C,D).

**Figure 4:**
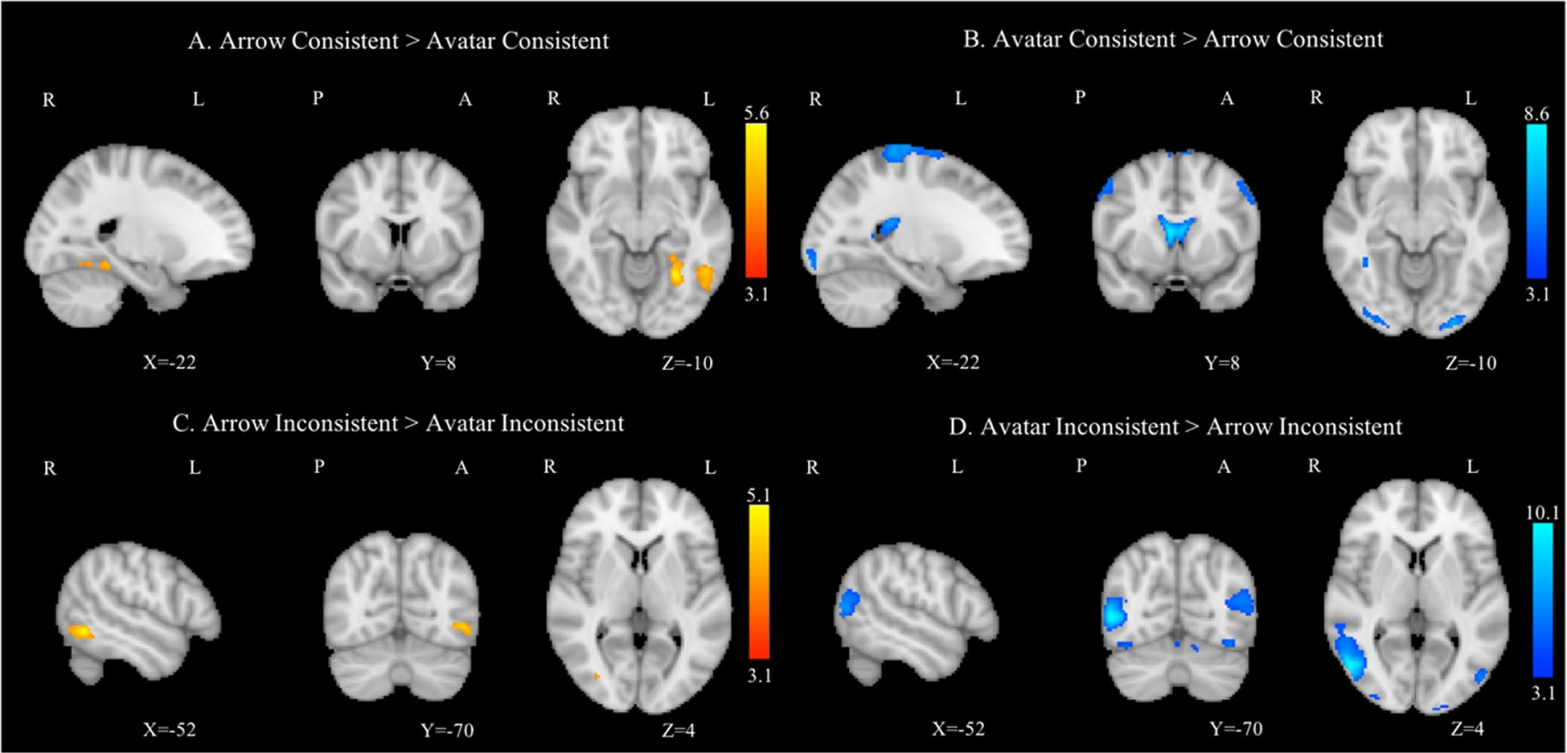
Group average task-related GLM analysis for the contrast Arrow Consistent > Avatar Consistent (**A**) and for the inverse comparison Avatar Consistent > Arrow Consistent (**B**), and for the contrasts Arrow Inconsistent > Avatar Inconsistent (**C**) and Avatar Inconsistent > Arrow Inconsistent (**D**). Right hemisphere on the left side, all clusters were thresholded by Z > 3.1 and a corrected cluster significance threshold of p = 0.05.

The comparison "Arrow Consistent versus Avatar Consistent" showed increased activation confined to the left lateral occipital and fusiform cortices. The inverse contrast generated an activation that concerned not only bilateral precentral gyrus, lateral occipital cortex and occipital pole but also the right fusiform cortex, right superior frontal gyrus and frontal pole (Fig. 4A,B). In the same line, the contrast "Arrow Inconsistent versus Avatar Inconsistent" revealed increased activation in the right lateral occipital cortex and the left inferior temporal gyrus. The inverse comparison displayed fMRI activation in the bilateral lateral occipital cortex, the left fusiform cortex and occipital pole, as well as the left frontal pole and paracingulate gyrus (Fig. 4C,D).

## Discussion

Our data reveal complex fMRI activation patterns during the performance of the level 1 Samson’s dPT and point to the parameters that should be taken into account when interpreting the neural substrates reflecting the ability to see the world from another person’s perspective. They first show that unlike non-mentalizing stimuli (Arrow), the recruitment of key ToM areas such as posterior cingulate cortex and precuneus is needed when treating human avatars. The successful adoption of other’s viewpoint relies on the activation of bilateral precuneus, left superior parietal cortex and posterior cingulate cortex (parts of the mentalizing networks) but also anterior cingulate cortex (salience network) and orbitofrontal cortex (decision making area). These findings support the idea that the integrity of ToM areas is necessary for the identification with anthropomorphic alter but not sufficient for sharing the other’s view, this latter depending on a complex interplay with other high-order cortical networks. Moreover, the present results provide the functional substrates of the dissociation between the implicit impact of other’s divergent viewpoint when we focus on our own visual experience and those related to the egocentric interference when judging other’s perspective.

There is still an ongoing debate regarding the mentalistic versus non-mentalistic brain activation during the performance of dPT. Ramsey et al.^15^ first reported that in dPT, the implicit computation of other’s perspectives takes place, which is independent of cognitive control. However, modifications of the dPT using transparent or opaque googles were inconclusive in respect to spontaneous perspective taking^11, 12^. Interestingly and somewhat counterintuitively, the mean reaction times were higher for Arrows than Avatars (without changes in performance) in our sample. This could be due to the additional effort needed due to the absence of familiarity facing Arrows or to the process of anthropomorphizing objects^20, 21^. Altercentric interference was equally present in Arrow and Avatar conditions supporting, at first glance, the idea of a domain-general attentional interpretation in dPT performances as suggested by Santiesteban et al.^13^. In a first fMRI study, Schurz et al.^16^ reported a spontaneous activation in right TPJ, ventral MPFC and ventral precuneus during self-perspective judgments when using an Avatar (mentalistic) but not an Arrow (non-mentalistic control) that was taken to reflect implicit processing of information linked to the other’s perspective during Self condition. This viewpoint has been challenged by neurostimulation reports, which show that transcranial magnetic stimulation of right TPJ impairs performance on all self-perspective trials (Arrow and Avatar), indicating the predominance of attentional processes rather than implicit mentalization^13, 22^. Based on the contrast between Arrow and Avatar conditions, our data provide evidence for a mixed scenario. The Arrow condition led to increased recruitment of two visual association areas, the bilateral lateral occipital and fusiform cortices. In the Avatar condition, besides the increased activation of bilateral occipital pole and the left lateral occipital cortex, we observed an increased recruitment of parts of the mentalizing network such as the posterior cingulate cortex and precuneus but also parts of the salience network such as the anterior cingulate cortex and right angular cortex. The latter is one of the main areas of the right TPJ known to be involved in most ToM paradigms^23^. Compared to the Arrow condition, increased activations were also observed in non-mentalistic areas such as the bilateral frontal pole for stimulus-dependent activation^24^ and the right supramarginal gyrus for attention reorienting^25^. The Arrow-Avatar/Consistent-Inconsistent coupling revealed differences and similarities in brain activation patterns. The fMRI activation patterns were similar in the presence of inconsistency for both Arrow and Avatar conditions with a main activation of central executive network areas. Most importantly, the direct comparison between Arrow Consistent versus Avatar Consistent and Arrow Inconsistent versus Avatar Inconsistent showed that in the presence of Avatar, brain activation was observed not only in primary and secondary visual areas but also in frontal lobe subdivisions and paracingulate gyrus. Altogether these results support the idea that mentalistic stimuli (Avatars) induce a distinct pattern of brain activation compared to non-mentalistic ones (Arrows), including parts of the mentalizing and salience networks and, to a lesser extent, attentional and cognitive control-related areas. Among mentalizing areas, activation was observed in posterior cingulate cortex and precuneus, two areas involved in imagery and imagination processes requiring the inference of mental states of others^26, 27^.

The Self-Other fMRI contrast produced some intriguing results. Self-perspective judgement was associated with increased recruitment of right superior and middle frontal gyrus that are part of the frontoparietal network involved in cognitive control and inhibition of irrelevant perspective when making visual perspective judgments^15, 28^. Additional activation was found also in the left angular cortex (for classical ToM areas) and supramarginal gyrus (attention reorienting). The inverse comparison revealed a more widespread activation of bilateral precuneus, left superior parietal cortex and posterior cingulate cortex^29, 30^. Precuneus activation is thought to occur in self-referential judgment, and first person perspective. Although its exact function in visual perspective taking is still matter of debate, its involvement in mental imagery, voluntary shifts in visual attention and consciousness implies that it may play a key role in the transformation of an egocentric frame of reference to other person’s perspective^31^. The left superior parietal cortex is engaged in perspective selection when choosing a relevant over an irrelevant perspective^32^. Moreover, two anterior areas showed higher BOLD signal in the Other condition, the anterior cingulate cortex, as part of the salience network, and orbitofrontal cortex, specialized in decision making. Taken together, these findings suggest that adopting the Self perspective in dPT needs primarily the activation of frontoparietal networks, while the Other perspective is a much more expensive brain process, and relies on the activation of classical ToM areas but also of salience network and decision making areas.

We report here a significant difference in reaction times for Self Inconsistent compared to Self Consistent conditions corresponding to the notion of altercentric interference. The significant prolongation of reaction times was also seen in Other Inconsistent compared to Other Consistent conditions corresponding to egocentric interference. By contrasting Self Consistent versus Self Inconsistent conditions, our fMRI observations provide new evidence about the functional background of altercentric interference. Only the left insula showed an increased activation in Self Consistent versus Inconsistent conditions. In contrast, Self Inconsistent condition was associated with a widespread activation of mentalizing and central executive areas. We found an increased activation of the bilateral angular gyrus, known to be a key ToM area (as part of the TPJ; for review see^17^) involved in self-other distinction^33^ but also bilateral inferior frontal gyri that are thought to be engaged whenever a task-relevant perspective needs to be selected over an irrelevant one^15^. In addition, facing the Self Inconsistent conditions and besides the expected activation of visual and attention orienting areas (lateral occipital cortex and right supramarginal gyrus), there is an increased activation of central executive areas as parts of the frontoparietal network, namely the superior and middle frontal cortex. These observations partly agree with those of Schurz and collaborators^16^ reporting an activation of right TPJ, and ventral medial PFC in condition of altercentric interference. Taken together, they support the role of a differentiated other-network that includes the angular gyrus and ventral medial PFC that allows for self-updating via integration of self-relevant information^34^. Using a visual perspective task, Martin et al.^19^ reported that excitatory high-definition transcranial direct current stimulation of the dorsomedial PFC enhanced the integration of external information into the Self whereas the opposite was true under inhibitory conditions. This area is known to be involved in self-other mergence and in particular in the estimation of others’ abilities as a function of our own judgement^35^. These findings reveal that altercentring interference is a complex phenomenon that needs the activation of brain networks involved in self-other distinction, estimation of the validity of others’ viewpoint, updating of self-relevant information, but also central executive functions.

In the Other Consistent condition, there was a restricted activation of mentalizing network components involved in visual perspective taking (precuneus, right medial frontal cortex^17, 23^) as well as salience network (anterior cingulate cortex). In the Other Inconsistent condition, we observed an activation in the bilateral lateral occipital cortex, precuneus cortex and superior parietal lobule, and in the middle, superior, precentral gyri and left frontal pole. The activation of precuneus irrespective of the consistency was reported by Schurz et al.^16^ but also by Sulpizio et al.^36^ and is thought to reflect the decentering in explicit perspective contrast rather than metalizing abilities per se. Importantly, our data show that the fMRI correlates of egocentric interference include parts of the mirror neuron system (middle and superior precentral gyri) as well as left frontopolar cortex known to be involved in deductive reasoning^37^. In particular, this latter area was associated with analogical reasoning and relational cognition, two cognitive processes involved in self-other distinction, decentering and inferential process^38^. Moreover, a recent EEG study showed that left frontopolar theta activity is associated with planning performance and successfully generated self-plans pointing to its role in the construction of self-centered cognitive processes^39^. These data imply that mentalization is not the main brain activity involved in Other Inconsistent trials. Egocentric interference seems to involve preferentially action understanding via the mirror neuron system and deductive reasoning. The absence of conflict in Other Consistent trials was associated with decreased recruitment of frontoparietal cortex whereas the opposite was true in both Self and Other Inconsistent trials^15^. Our observations confirm this viewpoint but also point to a more complex scenario involving mentalizing and salience networks as well as mirror neuron system as a function of the self-other focus in inconsistent trials. Importantly, previous studies indicated that dorsolateral PFC as well as more posterior and dorsal parts of the frontal cortex and TPJ may also be involved in consistency contrast for other perspective (for review see^18^). However, most of these studies used EEG or transcranial direct current stimulation in healthy controls, whereas the fMRI data were mostly obtained in clinical samples. Our observations in healthy controls do not support a preferential activation of these areas in the context of egocentric interference.

In conclusion, our data provide three new insights into the complex activation patterns in visual perspective taking. First, they support the idea that visual perspective taking in daily living is a highly specific process that needs the activation not only of the posterior cingulate cortex and precuneus but also parts of the salience network including the anterior cingulate and right angular cortex. In addition to the recruitment of visual association areas when using a non-mentalistic stimulus (Arrow), comparison to a human Avatar implies the activation of anterior cortical areas involved in emotional processing and theory of mind. Second, they indicate that, in terms of brain activation, “seeing it your way” has an increasing cost since it needs the recruitment of classical ToM areas but also decision making areas such as the orbitofrontal cortex in the Other compared to Self-condition. This observation may be particularly relevant when studying the decrease of ToM abilities reported in old age or in some psychiatric conditions known to affect these brain areas^40–42^. Whether or not mentalization is a key cognitive process in this contrast remains controversial. For some authors, explicit perspective contrast in level-1 visual perspective task is present both in Self and Other condition and may be cancelled after fMRI cognitive subtraction leaving out at least partly the mentalizing process. According to this perception, the observed activation patterns correspond mainly to self-other distinction (for review see^18^). The third and most important finding of this work concerns the identification of distinct fMRI correlates for egocentric and altercentric interference, two key notions in ToM research. The unconscious impact of other’s divergent viewpoint when we focus on our own visual experience is mostly driven by the activation of brain areas involved in self-other distinction (bilateral angular gyrus), self-updating via integration of self-relevant information (inferior frontal gyrus) but also central executive functions (superior and middle frontal cortex). Egocentric interference that contaminates our judgment of other’s perspective in inconsistent trials seems to follow different rules since it involves the activation of the mirror neuron system and deductive reasoning, much less related to pure ToM abilities. Based on these findings, one could expect that this interference would persist longer during the lifespan but also in clinical samples. Future studies in this field are warranted to test the validity of this hypothesis.

The strengths of the present data include the relatively large sample, and the separate analysis of the contrasts for the three main components of the dPT (Arrow versus Avatar, Self versus Other and Consistent versus Inconsistent). It is, however, important to define the limits of the present observations within the theoretical framework of ToM paradigms. The dPT is based on the judgment of visually presented situations. It requires an attribution of transient mental states without need for decoupling representations (dot arrangements and not only dot numbers), propositional content (in particular thoughts and beliefs), and overt action. In both Self and Other conditions, a task-relevant perspective needs to be selected over an irrelevant one, implying representation of the mental state of seeing and subsequent suppression of one’s own perspective. This description shows that there is an overlap with classical ToM paradigms, yet the dPT refers to the assessment of observation and inhibition of representation rather than that of beliefs, desires and resulting emotions. This difference should be taken into account when interpreting the fMRI correlates observed in our study. In the same line, mentalization is not a unique process and its characteristics vary substantially according to the experimental design so that the present observations do not allow for drawing general conclusions about the involvement of mentalizing networks in visual perspective tasks. Second, the fMRI activation patterns concern only men who were included in this study according to its initial design that has been made with focus on psychopathy in the context of forensic psychiatry. Third, from a strictly methodological viewpoint, in Consistent conditions, the dots are always illustrated in one hemifield, while for Inconsistent conditions there were both unilateral and bilateral trials. We cannot thus formally exclude that differences in activation in visual/parietal areas may partly reflect a larger search area for the dots displayed in both hemifields. However, this is an unlikely scenario for several reasons. The actual visual stimulation of the dot is relatively low (with respect to the total visual processing and presentation), regardless whether unilateral or bilateral. While the participant performs the task, there is no prior instruction whether the subsequent condition will be Consistent or Inconsistent. Subsequently, in both conditions, the participant will have to examine the entire scene (both hemifields) decreasing the potential bias related to visual search area. Lastly, the number of unilateral trials (n = 95 for the 4 runs) exceeded by far that of bilateral (n = 33 for the four runs), indicating that the effect of visual search area, if any, should be of low range in our study. Fourth, no control was included for possible confounds such as global intelligence, and attention performance that could critically affect performance in dPT. Fifth, non ToM-related interpretations for the patterns of brain activation observed in Self Consistent versus Self Inconsistent and Other Consistent versus Other Inconsistent conditions could be considered. For instance, most of the areas activated by inconsistency in both conditions partly overlap with the functional regions of the supplementary eye field, bilateral frontal and parietal eye fields. It is thus possible that the detection of inconsistent trials may also be more challenging and needs exact visual analysis implying a higher activation in eye movement coordination centers. Sixth, the associations involving the TPJ should be considered with caution since this areas is highly heterogeneous according to the atlas modalities used for its definition (gyral, sulco-gyral, cytoarchitectonic, connectivity-based^25^). Given the TPJ is an a priori region of interest for visual perspective taking, it is noteworthy that the activation listed as lateral occipital cortex includes a number of voxels that would fit most characterizations of TPJ anatomically. Last but not least, the present findings concern only the Samson’s dPT and are not applicable to more complex ToM paradigms but also level 2 visual perspective tasks involving how the objects and their arrangement may look to another person. Future studies in larger samples comparing level 1 and level 2 tasks taking into account the abovementioned confounds in healthy adults but also clinical populations (such those described by Drayton et al. in the study of psychopaths using the dPT^43^) would be of great interest to obtain further insight into the complex puzzle of brain activation related to perspective taking.

## Methods

### Ethical statements

The study was reviewed and approved by the local Ethics Committee [Commission cantonale d’éthique de la recherche (CCER)]. The participants provided their written informed consent prior to inclusion. All the cases were recruited via advertisements in local newspapers and media. All methods were performed in accordance with the World Medical Association Declaration of Helsinki and the principles of Good Clinical Practice.

### Participants

The study was approved by the local Ethics Committee and all participants gave written informed consent prior to inclusion. All the cases were recruited via advertisements in local newspapers and media. The present sample included 82 community-dwelling men (mean age 32.7 ± 11.4 years, range age 19–66 years). All participants performed a neurocognitive assessment. Subjects with a history of a chronic psychiatric disorder (psychosis or bipolar disorder), loss of consciousness lasting longer than 30 min, head injury or post-concussion symptoms, auditory or visual deficits, seizure and neurological disorders, and regular use of psychotropic medications were excluded. Structural brain abnormalities were excluded after routine radiological assessments.

### Computer-based response-time task of automatic ToM

**Figure 5:**
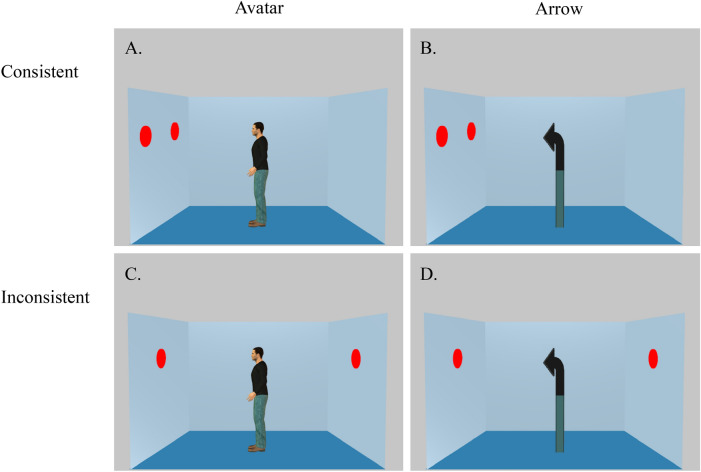
Examples of the stimuli presented in the different experimental conditions: Consistent (**A**) and Inconsistent (**C**) conditions with the Avatar, and Consistent (**B**) and Inconsistent (**D**) conditions with the Arrow.

We used an adapted computer-based response-time task developed by Samson and colleagues^8^ (Experiment 1). The stimuli consisted of a picture showing a lateral view into a room with the left, back, and right walls visible. Red discs were displayed on one or two walls. A human avatar or an arrow, which had the same characteristics as the human avatar in terms of color palette and distribution as well as height and surface, always appeared in the center of the room in profile facing either the right or the left wall. Depending upon the orientation of the avatar or of the arrow and the positioning of the discs, the avatar or the arrow was able or unable to see all the discs in the room. On each trial, participants judged either their own visual perspective (Self trials) or the visual perspective of the avatar/arrow (Avatar/Arrow trials) (Fig. 5). Specifically, participants were asked to verify the number of discs that either they (Self) or the avatar/arrow could see. On 50% trials, the participant and the avatar/arrow could see the same number of discs (Consistent perspective condition). On 50% trials, they could see a different numbers of discs (Inconsistent perspective condition). The position of the Avatar/Arrow was kept constant across consistent and inconsistent trials, but the position of the discs changed. Each trial included four stimuli, presented in the center of the screen in the following order: (i) a fixation cross indicating the start of the trial, (ii) a word indicating whether participants should adopt their own perspective (“YOU”) or the perspective of the avatar (“HE”) or of the arrow (“IT”), (iii) a number (0–3) specifying the content to be verified, and (iv) a picture of the avatar/arrow in a room. Stimuli i–iii each appeared for 750 ms, and each was followed by a blank screen for 500 ms. After the final stimulus, participants had 2000 ms to indicate whether the picture matched the specified perspective and content (“yes” response), or that it did not match the specified perspective and content (“no” response). The next trial was delivered after 2000 ms if no response was given. Participants did not receive any trial-by-trial feedback about their performance. Trials were presented in four blocks, each consisting of 36 trials. Each block also included 4 filler trials in which there were no discs on the walls of the room. These filler trials were included to ensure that the correct response to the perspective “YOU” and perspective content “0” could sometimes be “yes.” The order of presentation of the blocks was randomized and counterbalanced across participants. The entire procedure was conducted using E-Prime 3.0 software to control the stimulus presentation and data collection (https://pstnet.com/products/e-prime/).

### MR imaging

MR imaging were acquired using a 3 T MRI scanner (MAGNETOM PRISMA, Siemens) at Campus Biotech Geneva (https://www.campusbiotech.ch/). Functional echo-planar imaging had the following essential parameters: 66 slices, slice thickness = 2.0 mm, voxel size = 2.0 × 2.0 × 2.0 mm^3^, repetition time = 1000 ms, echo time = 32 ms, flip angle = 50°, field of view = 224 mm, resulting in 8.05 min per fMRI run. Each participant performed the 4 runs in a pseudo-randomized design. An additionally acquired 3DT1 sequence (208 slices; slice thickness = 1.0 mm; voxel size = 1 × 1 × 1 mm^3^; repetition time = 2300 ms; echo time = 2.26 ms; flip angle = 8°; field of view = 256 mm) was used for spatial normalization and registration.

### Statistics

#### Behavioral data

Anticipatory responses (< 200 ms) or delayed responses (> 2000 ms) were counted as errors. The response times were normally distributed as assessed by Shapiro–Francia tests. To explore the association between reaction times and the three experimental conditions (Arrow vs Avatar, Self vs Other, Consistent Inconsistent) we used multiple linear mixed regression models with and without interaction terms. To explore the association between the occurrence of errors and the three experimental conditions we used multiple logistic mixed regression models with and without interaction terms. In addition, the same simple mixed regression models were used to assess the differences between the experimental conditions.

#### GLM analyses of task-related activation

Task-related GLM data processing was carried out using FEAT (FMRI Expert Analysis Tool) Version 6.0.2, part of FSL (FMRIB’s Software Library, www.fmrib.ox.ac.uk/fsl). At the first level, we performed a within-session analysis. At the second level, we input the data from Level 1 and estimated each participant’s mean response. At the third level, the group across all 82 participants was calculated. Higher-level analysis was carried out using a mixed effects model, by forcing the random effects variance to zero in FLAME (FMRIB’s Local Analysis of Mixed Effects)^44–46^. Z (Gaussianised T/F) statistic images were thresholded using clusters determined by Z > 3.1 and a corrected cluster significance threshold of p = 0.05^47^. Anatomic location of the activation clusters was determined using ‘‘atlasquery’’, part of FSL, and the Harvard–Oxford Cortical Structural Atlas.

## Supplementary Information

Supplementary Tables.

## Data Availability

The data are available from the corresponding author upon reasonable request.
