# Supplementary material for: Patterns of multiple brain network activation in dot perspective task
Source: Sci Rep. 2023 Apr 26;13:6793. doi: 10.1038/s41598-023-33427-1 (PMC10133244; doi:10.1038/s41598-023-33427-1)
Supplement: Supplementary file 1 — Supplementary Tables. [file 41598_2023_33427_MOESM1_ESM.docx]

Table 1. GLM results: Arrow > Avatar

| Cluster Index | Voxels | P | -log10(P) | Z-MAX | Z-MAX X (mm) | Z-MAX Y (mm) | Z-MAX Z (mm) | Side | Regions |
| --- | --- | --- | --- | --- | --- | --- | --- | --- | --- |
| 1 | 709 | 2.38e-06 | 7.62 | 6.03 | -52 | -60 | -8 | L | Inferior Temporal Gyrus |
| 2 | 231 | 0.00116 | 2.93 | 5.79 | 26 | -40 | -16 | R | Temporal Occipital Fusiform Cortex |
| 3 | 186 | 0.00429 | 2.37 | 5.42 | 34 | -78 | 10 | R | Lateral Occipital Cortex, inf. division |
| 4 | 159 | 0.00984 | 2.01 | 5.36 | -34 | -84 | 8 | L | Lateral Occipital Cortex, sup. division |

Table 2. GLM results: Avatar > Arrow

| Cluster Index | Voxels | P | -log10(P) | Z-MAX | Z-MAX X (mm) | Z-MAX Y (mm) | Z-MAX Z (mm) | Side | Regions |  |
| --- | --- | --- | --- | --- | --- | --- | --- | --- | --- | --- |
| 1 | 3815 | 8.66e-27 | 26.1 | 5,26 | -18 | -8 | 74 | L | Superior Frontal Gyrus | |
| 2 | 3272 | 3.96e-24 | 23.4 | 11.3 | 40 | -46 | -18 | R | Temporal Occipital Fusiform Cortex | |
| 3 | 890 | 8.31e-10 | 9.08 | 7.82 | -50 | -76 | 12 | L | Lateral Occipital Cortex, inf. division | |
| 4 | 582 | 2.98e-07 | 6.53 | 4.95 | 24 | 38 | 40 | R | Frontal Pole | |
| 5 | 531 | 8.94e-07 | 6.05 | 4.89 | -26 | 50 | 38 | L | Frontal Pole | |
| 6 | 442 | 6.08e-06 | 5.22 | 4.81 | 0 | -50 | 48 | L | Precuneus Cortex | |
| 7 | 377 | 2.74e-05 | 4.56 | 7.16 | -28 | -94 | -12 | L | Occipital Pole | |
| 8 | 239 | 0.000931 | 3.03 | 5.11 | -38 | -68 | -16 | L | Occipital Fusiform Gyrus | |
| 9 | 194 | 0.00338 | 2.47 | 4.65 | -8 | -12 | 22 | L | Lateral Ventricle | |
| 10 | 144 | 0.0159 | 1.8 | 4.69 | -2 | -74 | -16 | L | Lingual Gyrus | |
| 11 | 132 | 0.0236 | 1.63 | 4.16 | 6 | -36 | -4 | R | Lingual Gyrus | |

Table 3. GLM results: Self > Other

| Cluster Index | Voxels | | P | | -log10(P) | Z-MAX | Z-MAX X (mm) | Z-MAX Y (mm) | | Z-MAX Z (mm) | | Side | | Regions | |  |
| --- | --- | --- | --- | --- | --- | --- | --- | --- | --- | --- | --- | --- | --- | --- | --- | --- |
| 1 | | 451 | | 8.82e-06 | 5.05 | 5.53 | -14 | | -82 | | -10 | | L | | Lingual Gyrus | |
| 2 | | 339 | | 0.000109 | 3.96 | 4.35 | -48 | | -50 | | 40 | | L | | Supramarginal Gyrus | |
| 3 | | 198 | | 0.00406 | 2.39 | 5.06 | 24 | | 24 | | 50 | | R | | Superior Frontal Gyrus | |
| 4 | | 139 | | 0.0234 | 1.63 | 3.91 | 14 | | -90 | | 26 | | R | | Occipital Pole | |

Table 4. GLM results: Other > Self

| Cluster Index | Voxels | P | -log10(P) | Z-MAX | Z-MAX X (mm) | Z-MAX Y (mm) | Z-MAX Z (mm) | Side | Regions |
| --- | --- | --- | --- | --- | --- | --- | --- | --- | --- |
| 1 | 3239 | 5.82e-23 | 22.2 | 7.44 | -38 | -44 | -18 | L | Temporal Occipital Fusiform Cortex |
| 2 | 2626 | 6.77e-20 | 19.2 | 5.82 | -2 | 44 | -12 | L | Frontal Medial Cortex |
| 3 | 1820 | 1.82e-15 | 14.7 | 7.86 | 36 | -86 | -4 | R | Lateral Occipital Cortex |
| 4 | 1297 | 3.11e-12 | 11.5 | 5.66 | 14 | -58 | 30 | R | Precuneus Cortex |
| 5 | 233 | 0.00155 | 2.81 | 4.58 | -6 | -22 | 36 | L | Posterior Cingulate Gyrus |
| 6 | 197 | 0.00418 | 2.38 | 4.8 | -34 | 26 | -16 | L | Frontal Orbital Cortex |
| 7 | 118 | 0.046 | 1.34 | 5.1 | 26 | -32 | 6 | R | Cerebral White Matter |

Table 5. GLM results: Arrow Inconsistent > Arrow Consistent

| Cluster Index | Voxels | P | -log10(P) | Z-MAX | Z-MAX X (mm) | Z-MAX Y (mm) | Z-MAX Z (mm) | Side | Regions |
| --- | --- | --- | --- | --- | --- | --- | --- | --- | --- |
| 1 | 1963 | 1.3e-16 | 15.9 | 5.77 | -54 | 20 | 30 | L | Middle Frontal Gyrus |
| 2 | 1464 | 1.45e-13 | 12.8 | 5.43 | 48 | 14 | 42 | R | Middle Frontal Gyrus |
| 3 | 1163 | 1.47e-11 | 10.8 | 4.94 | 36 | -52 | 46 | R | Superior Parietal Lobule |
| 4 | 1048 | 9.54e-11 | 10 | 5.29 | -34 | -44 | 42 | L | Superior Parietal Lobule |
| 5 | 375 | 3.78e-05 | 4.42 | 6.47 | 6 | 10 | 12 | R | Lateral Ventricle |
| 6 | 335 | 9.78e-05 | 4.01 | 4.28 | -4 | 32 | 44 | L | Superior Frontal Gyrus |
| 7 | 141 | 0.0199 | 1.7 | -4.04 | -42 | 50 | 2 | L | Frontal Pole |

Table 6. GLM results: Avatar Consistent > Avatar Inconsistent

| Cluster Index | Voxels | P | -log10(P) | Z-MAX | Z-MAX X (mm) | Z-MAX Y (mm) | Z-MAX Z (mm) | Side | Regions |
| --- | --- | --- | --- | --- | --- | --- | --- | --- | --- |
| 1 | 1457 | 7.29e-14 | 13.1 | 6.7 | -30 | -44 | 6 | L | Cerebral White Matter, Lateral Ventricle |

Table 7. GLM results: Avatar Inconsistent > Avatar Consistent

| Cluster Index | Voxels | P | -log10(P) | Z-MAX | Z-MAX X (mm) | Z-MAX Y (mm) | Z-MAX Z (mm) | Side | Regions |
| --- | --- | --- | --- | --- | --- | --- | --- | --- | --- |
| 1 | 2164 | 3.24e-18 | 17.5 | 6.41 | 32 | -44 | 46 | R | Superior Parietal Lobule |
| 2 | 1682 | 2.59e-15 | 14.6 | 5.96 | -32 | -50 | 48 | L | Superior Parietal Lobule |
| 3 | 314 | 0.000126 | 3.9 | 5.51 | -44 | 6 | 34 | L | Middle Frontal Gyrus |
| 4 | 191 | 0.00364 | 2.44 | 4.23 | -50 | -52 | -4 | L | Middle Temporal Gyrus |
| 5 | 155 | 0.0111 | 1.96 | 4.69 | -34 | 4 | 46 | L | Middle Frontal Gyrus |
| 6 | 142 | 0.0168 | 1.77 | 5.19 | -6 | 18 | 52 | L | Superior Frontal Gyrus |

Table 8. GLM results: Self Consistent > Self Inconsistent

| Cluster Index | | Voxels | P | | | -log10(P) | Z-MAX | Z-MAX X (mm) | | Z-MAX Y (mm) | | Z-MAX Z (mm) | Side | | Regions |
| --- | --- | --- | --- | --- | --- | --- | --- | --- | --- | --- | --- | --- | --- | --- | --- |
| 1 | 212 | | | 0.00212 | 2.67 | | 4.36 | -36 | -4 | | 0 | | L | Insular Cortex | |

Table 9. GLM results: Self Inconsistent > Self Consistent

| Cluster Index | Voxels | P | -log10(P) | Z-MAX | Z-MAX X (mm) | Z-MAX Y (mm) | Z-MAX Z (mm) | Side | | Regions |  |
| --- | --- | --- | --- | --- | --- | --- | --- | --- | --- | --- | --- |
| 1 | 1271 | 1.74e-12 | 11.8 | 5.69 | -56 | 20 | 8 | | L | Inferior Frontal Gyrus | |
| 2 | 767 | 9.26e-09 | 8.03 | 5.06 | -4 | 32 | 52 | | L | Superior Frontal Gyrus | |
| 3 | 488 | 2.44e-06 | 5.61 | 5.06 | 6 | 12 | 8 | | R | Caudate | |
| 4 | 473 | 3.4e-06 | 5.47 | 4.32 | 40 | -42 | 42 | | R | Supramarginal Gyrus | |
| 5 | 431 | 8,64e-06 | 5.06 | 4.23 | 42 | 8 | 56 | | R | Middle Frontal Gyrus | |
| 6 | 366 | 3.91e-05 | 4.41 | 4.66 | -38 | -52 | 48 | | L | Superior Parietal Lobule | |

Table 10. GLM results: Other Consistent > Other Inconsistent

| Cluster Index | Voxels | P | -log10(P) | Z-MAX | Z-MAX X (mm) | Z-MAX Y (mm) | Z-MAX Z (mm) | Side | | Regions | |
| --- | --- | --- | --- | --- | --- | --- | --- | --- | --- | --- | --- |
| 1 | 586 | 4.17e-07 | 6.38 | 4.76 | 6 | 20 | -6 | | R | Caudate |  |
| 2 | 240 | 0.00108 | 2.97 | 4.18 | -10 | -54 | 12 | | L | Precuneus |  |
| 3 | 156 | 0.0122 | 1.91 | 4.14 | -8 | -96 | 24 | | L | Occipital Pole |  |

Table 11. GLM results: Other Inconsistent > Other Consistent

| Cluster Index | Voxels | P | -log10(P) | Z-MAX | Z-MAX X (mm) | Z-MAX Y (mm) | Z-MAX Z (mm) | Side | | Regions | | |
| --- | --- | --- | --- | --- | --- | --- | --- | --- | --- | --- | --- | --- |
| 1 | 6079 | 5.47e-36 | 35.3 | 7.01 | -32 | -50 | 48 | | L | | Superior Parietal Lobule |  |
| 2 | 3085 | 1.06e-22 | 22 | 7.07 | -44 | 6 | 34 | | L | | Middle Frontal Gyrus |  |
| 3 | 1736 | 2.67e-15 | 14.6 | 5.39 | 42 | 12 | 30 | | R | | Middle Frontal Gyrus |  |
| 4 | 467 | 4.65e-06 | 5.33 | 5.16 | -4 | 30 | 46 | | L | | Superior Frontal Gyrus |  |
| 5 | 447 | 7.21e-06 | 5.14 | 4.77 | -54 | -58 | -12 | | L | | Inferior Temporal Gyrus |  |

Table 12. GLM results: Arrow Consistent > Avatar Consistent

| Cluster Index | Voxels | P | -log10(P) | Z-MAX | Z-MAX X (mm) | Z-MAX Y (mm) | Z-MAX Z (mm) | | Side | | Regions | | |
| --- | --- | --- | --- | --- | --- | --- | --- | --- | --- | --- | --- | --- | --- |
| 1 | 367 | 4.19e-05 | 4.38 | 5.25 | -44 | -64 | | -4 | | L | | Lateral Occipital Cortex |  |
| 2 | 276 | 0.000398 | 3.4 | 5.61 | -28 | -56 | | -12 | | L | | Temporal Occipital Fusiform Cortex |  |

Table 13. GLM results: Avatar Consistent > Arrow Consistent

| Cluster Index | Voxels | P | -log10(P) | Z-MAX | Z-MAX X (mm) | Z-MAX Y (mm) | | Z-MAX Z (mm) | | Side | | Regions | | |
| --- | --- | --- | --- | --- | --- | --- | --- | --- | --- | --- | --- | --- | --- | --- |
| 1 | 2207 | 3.88e-18 | 17.4 | 8.66 | 6 | | 10 | | 12 | | R | | Lateral Ventricle |  |
| 2 | 1947 | 1.22e-16 | 15.9 | 6.22 | -16 | | -12 | | 76 | | L | | Precentral Gyrus |  |
| 3 | 900 | 9.95e-10 | 9 | 7.01 | 52 | | -70 | | 2 | | R | | Lateral Occipital Cortex |  |
| 4 | 459 | 5.19e-06 | 5.29 | 4.9 | 58 | | 16 | | 34 | | R | | Precentral Gyrus |  |
| 5 | 347 | 6.75e-05 | 4.17 | 8.24 | 40 | | -46 | | -18 | | R | | Temporal Occipital Fusiform Cortex |  |
| 6 | 324 | 0.000118 | 3.93 | 4.8 | 14 | | 52 | | 48 | | R | | Frontal Pole |  |
| 7 | 278 | 0.000038 | 3.42 | 6.5 | -48 | | -76 | | 6 | | L | | Lateral Occipital Cortex |  |
| 8 | 204 | 0.00283 | 2.55 | 5.83 | -28 | | -94 | | -12 | | L | | Occipital Pole |  |
| 9 | 199 | 0.00327 | 2.49 | 3.98 | 4 | | 16 | | 56 | | R | | Superior Frontal Gyrus |  |
| 10 | 142 | 0.0185 | 1.73 | 4.91 | 28 | | -94 | | -4 | | R | | Occipital Pole |  |
| 11 | 136 | 0.0225 | 1.65 | 4.05 | -56 | | 10 | | 38 | | L | | Precentral Gyrus |  |

Table 14. GLM results: Arrow Inconsistent > Avatar Inconsistent

| Cluster Index | Voxels | P | -log10(P) | Z-MAX | Z-MAX X (mm) | Z-MAX Y (mm) | | Z-MAX Z (mm) | | Side | | Regions | | |
| --- | --- | --- | --- | --- | --- | --- | --- | --- | --- | --- | --- | --- | --- | --- |
| 1 | 222 | 0.00126 | 2.9 | 5.11 | -52 | | -60 | | -8 | | L | | Inferior Temporal Gyrus |  |
| 2 | 147 | 0.0128 | 1.89 | 5.17 | 36 | | -80 | | 8 | | R | | Lateral Occipital Cortex |  |

Table 15. GLM results: Avatar Inconsistent > Arrow Inconsistent

| Cluster Index | Voxels | P | -log10(P) | Z-MAX | Z-MAX X (mm) | Z-MAX Y (mm) | | Z-MAX Z (mm) | | Side | | Regions | | |
| --- | --- | --- | --- | --- | --- | --- | --- | --- | --- | --- | --- | --- | --- | --- |
| 1 | 2642 | 2.66e-21 | 20.6 | 10.1 | 52 | | -70 | | 4 | | R | | Lateral Occipital Cortex |  |
| 2 | 573 | 2.38e-07 | 6.62 | 6.47 | -50 | | -76 | | 12 | | L | | Lateral Occipital Cortex |  |
| 3 | 228 | 0.00106 | 2.97 | 4.12 | -28 | | 50 | | 36 | | L | | Frontal Pole |  |
| 4 | 221 | 0.0013 | 2.89 | 5.88 | -28 | | -94 | | -12 | | L | | Occipital Pole |  |
| 5 | 211 | 0.00174 | 2.76 | 5.61 | -38 | | -54 | | -18 | | L | | Temporal Occipital Fusiform Cortex |  |
| 6 | 126 | 0.026 | 1.59 | 4.62 | -6 | | 10 | | 44 | | L | | Paracingulate Gyrus |  |
| 7 | 116 | 0.0368 | 1.43 | 4.26 | -6 | | -74 | | -20 | | L | | Occipital Fusiform Gyrus |  |

Table 16. GLM results: Self Inconsistent (Avatar) > Self Consistent (Avatar)

| Cluster Index | Voxels | P | -log10(P) | Z-MAX | Z-MAX X (mm) | Z-MAX Y (mm) | | Z-MAX Z (mm) | | Side | | Regions | | |
| --- | --- | --- | --- | --- | --- | --- | --- | --- | --- | --- | --- | --- | --- | --- |
| 1 | 11976 | 0 | 57.6 | 7.84 | 2 | | 42 | | 40 | | R | | Superior Frontal Gyrus |  |
| 2 | 4289 | 3.96e-29 | 28.4 | 6.65 | 46 | | -40 | | 56 | | R | | Supramarginal Gyrus |  |
| 3 | 2991 | 8.69e-23 | 22.1 | 6.34 | -34 | | -50 | | 46 | | L | | Superior Parietal Lobule |  |
| 4 | 2710 | 2.66e-21 | 20.6 | 8.52 | -4 | | 10 | | 10 | | L | | Lateral Ventricle, Left Caudate |  |
| 5 | 1292 | 8.8e-13 | 12.1 | 5.53 | -50 | | -62 | | -10 | | L | | Inferior Temporal Gyrus |  |
| 6 | 489 | 2.03e-06 | 5.69 | 4.98 | 56 | | -48 | | -10 | | R | | Middle Temporal Gyrus |  |
| 7 | 424 | 8.64e-06 | 5.06 | 4.86 | -4 | | -44 | | -2 | | L | | Cingulate Gyrus |  |
| 8 | 284 | 0.000267 | 3.57 | 4.74 | 18 | | -96 | | -8 | | R | | Occipital Pole |  |
| 9 | 240 | 0.000874 | 3.06 | 5.43 | 18 | | -34 | | 80 | | R | | Postcentral Gyrus |  |
| 10 | 183 | 0.00456 | 2.34 | 4.53 | 40 | | 18 | | -16 | | R | | Frontal Orbital Cortex |  |

Table 17. GLM results: Self Consistent (Arrow) > Self Inconsistent (Arrow)

| Cluster Index | Voxels | P | -log10(P) | Z-MAX | Z-MAX X (mm) | Z-MAX Y (mm) | | Z-MAX Z (mm) | | Side | | Regions | | |
| --- | --- | --- | --- | --- | --- | --- | --- | --- | --- | --- | --- | --- | --- | --- |
| 1 | 3374 | 7.23e-26 | 25.1 | 5.33 | -10 | | -46 | | 72 | | L | | Postcentral Gyrus |  |
| 2 | 2071 | 1.61e-18 | 17.8 | 5.08 | 48 | | -8 | | -8 | | R | | Planum Polare |  |
| 3 | 1792 | 9.21e-17 | 16 | 5.6 | -16 | | -4 | | -4 | | L | | Left Pallidum |  |
| 4 | 1478 | 1.14e-14 | 13.9 | 5.65 | -42 | | -2 | | -2 | | L | | Insular Cortex |  |
| 5 | 395 | 9.6e-06 | 5.02 | 4.36 | -4 | | 8 | | 8 | | L | | Lateral Ventricle, Caudate |  |
| 6 | 380 | 1.39e-05 | 4.86 | 5.48 | 4 | | 18 | | 18 | | R | | Cingulate Gyrus |  |
| 7 | 183 | 0.00333 | 2.48 | 4.2 | 22 | | -8 | | -8 | | R | | Cerebral White Matter |  |
| 8 | 117 | 0.0312 | 1.51 | 4.47 | 6 | | 28 | | 28 | | R | | Cingulate Gyrus |  |
| 9 | 114 | 0.0348 | 1.46 | 4.63 | -14 | | 10 | | 10 | | L | | Caudate |  |

Table 18. GLM results: Self Inconsistent (Arrow) > Self Inconsistent (Avatar)

| Cluster Index | Voxels | P | -log10(P) | Z-MAX | Z-MAX X (mm) | Z-MAX Y (mm) | | Z-MAX Z (mm) | | Side | | Regions | | |
| --- | --- | --- | --- | --- | --- | --- | --- | --- | --- | --- | --- | --- | --- | --- |
| 1 | 927 | 2.6e-10 | 9.58 | 6.5 | 36 | | -80 | | 10 | | R | | Lateral Occipital Cortex |  |
| 2 | 132 | 0.0211 | 1.68 | 4.82 | -28 | | -48 | | -14 | | L | | Temporal Occipital Fusiform Cortex |  |

Table 19. GLM results: Self Inconsistent (Avatar) > Self Inconsistent (Arrow)

| Cluster Index | Voxels | P | -log10(P) | Z-MAX | Z-MAX X (mm) | Z-MAX Y (mm) | | Z-MAX Z (mm) | | Side | | Regions | | |
| --- | --- | --- | --- | --- | --- | --- | --- | --- | --- | --- | --- | --- | --- | --- |
| 1 | 1093 | 1.42e-11 | 10.8 | 4.66 | 0 | | 54 | | 32 | | L | | Superior Frontal Gyrus |  |
| 2 | 947 | 1.82e-10 | 9.74 | 7.43 | 52 | | -68 | | 4 | | R | | Lateral Occipital Cortex |  |
| 3 | 899 | 4.32e-10 | 9.36 | 5.22 | -54 | | -70 | | 12 | | L | | Lateral Occipital Cortex |  |
| 4 | 516 | 8.34e-07 | 6.08 | 4.81 | -8 | | 2 | | 16 | | L | | Lateral Ventricle, Caudate |  |
| 5 | 354 | 3.71e-05 | 4.43 | 4.42 | 0 | | -68 | | 48 | | R | | Precuneus Cortex |  |
| 6 | 315 | 9.95e-05 | 4 | 7.38 | 40 | | -46 | | -18 | | R | | Temporal Occipital Fusiform Cortex |  |
| 7 | 170 | 0.00607 | 2.22 | 4.5 | -48 | | 36 | | 2 | | L | | Inferior Frontal Gyrus |  |
| 8 | 149 | 0.0119 | 1.92 | 3.83 | -56 | | 16 | | 30 | | L | | Inferior Frontal Gyrus |  |

Table 20. GLM results: (Arrow Inconsistent - Arrow Consistent) > (Avatar Inconsistent - Avatar Consistent)

| Cluster Index | Voxels | P | -log10(P) | Z-MAX | Z-MAX X (mm) | Z-MAX Y (mm) | | Z-MAX Z (mm) | | Side | | Regions | | |
| --- | --- | --- | --- | --- | --- | --- | --- | --- | --- | --- | --- | --- | --- | --- |
| 1 | 1436 | 1.02e-13 | 13 | 8.18 | 6 | | 10 | | 12 | | R | | Lateral Ventricle, Caudate |  |
